# Supplementary material for: Crystal Structure of a Classical MHC Class I Molecule in Dogs; Comparison of DLA-88*0 and DLA-88*5 Category Molecules
Source: Cells. 2023 Apr 6;12(7):1097. doi: 10.3390/cells12071097 (PMC10093629; doi:10.3390/cells12071097)

Figure S1. Comparison of the folding of the main chain of peptide ligand RTI9 in pDLA-88*001:04 with that of peptides in other pMHC-I complexes

The present study determined the structure of RTI9/DLA-88*001:04/β2m (PDB accession 7CJQ). In (A) the binding of the RTI9 peptide (RTISYTYPF) in the PBG of this structure is shown from above. In (B), superimposition of various pMHC-I structures shows that the RTI9 peptide main chain (magenta) forms an “M” structure, similar but not identical to the peptide folding in several other pMHC-I structures (codes before the commas are PDB accession numbers): 3QQ3, swine Sla-1 0401; 1VGK, mouse H-2kd; 3OXS, HLA-A*02:07; 3PWU, cattle Bola-A11; 3RWC, rhesus macaque Mamu-B17-Iw9; 5Y91, grass carp UAA106; 5F1I, dog DLA-88*508:01. The peptide ligands in (B) are shown from the side in ribbon format.

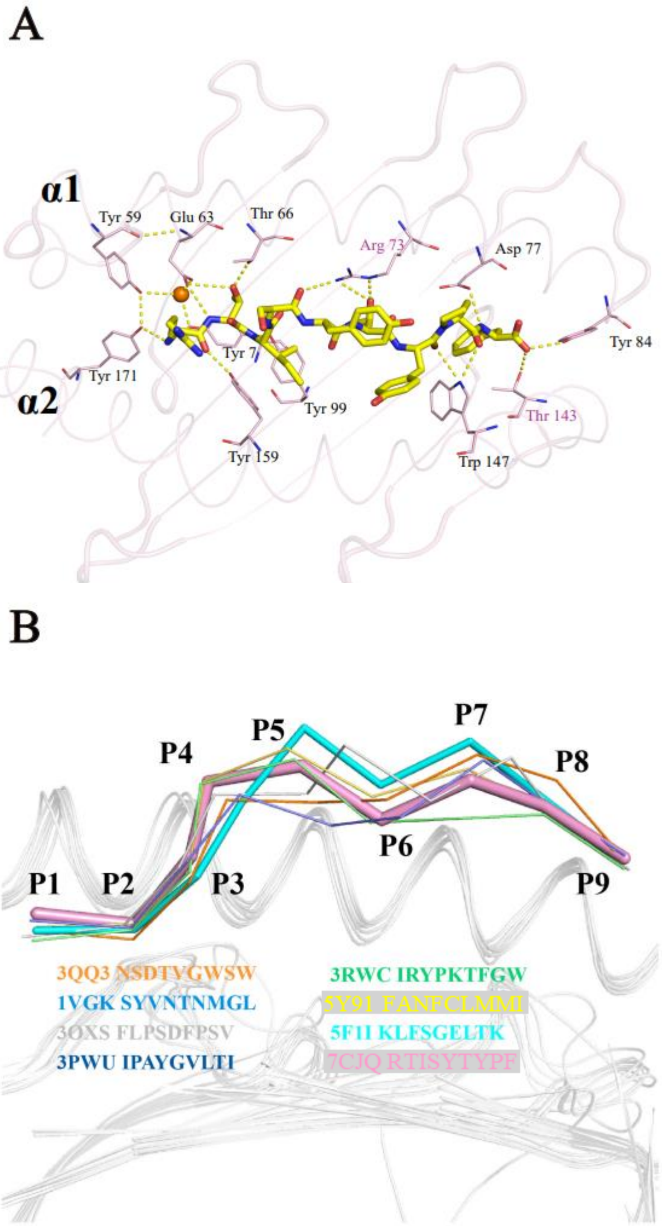

Supplement: Supplementary file 1 [file cells-12-01097-s001.zip › Figure S1.pdf]
