# Supplementary material for: Crystal Structure of a Classical MHC Class I Molecule in Dogs; Comparison of DLA-88*0 and DLA-88*5 Category Molecules
Source: Cells. 2023 Apr 6;12(7):1097. doi: 10.3390/cells12071097 (PMC10093629; doi:10.3390/cells12071097)
Supplement: Supplementary file 1 [file cells-12-01097-s001.zip › Table S1 revised.pdf]

Table S1. Predicted peptides and their binding to DLA-88*001:04/β2m evaluated by *in vitro* refolding

Derived protein[a]	name	Sequence	Stability[b]
Rabies-virus-	MTT9	MTTKSVSFR	++
glycoprotein	VSF9	VSFRRLSHL	++
H5N1-H	YNA9	YNAELLVLM	++
H7N9-NA	DGA9	DGAKIEYFL	--
H7N9-H	VAM9	VAMENQHTI	++
Canine-self	KLF9	KLFSGELTK	++
CDV-H	VVL9	VVLPTQNFR	++
	ITN9	ITNHRGFLK	++
	TSV9	TSVGRFFPL	++
	RTI9	RTISYTYPF	++
	TSV9	TSVGRVFPL	++
	RVF9	RVFEIGFIK	--
CDV-N	FSA9	FSAGSYPLL	++
	YPA9	YPALGLHEF	++
	EAG9	EAGLASFIL	+-
CPV-VP1	PSN9	PSNIGGMKI	+-
	TNP9	TNPSDAAAK	--
CPV-NS1	MSG9	MSGNQYTEE	--

[a]H5N1 and H7N9 are influenza virus subtypes; CDV, canine distemper virus; CPV, canine parvovirus; H, hemagglutinin; NA, neuraminidase; N, nucleocapsid; VP1 is a capsid protein; NS1, nonstructural protein 1

[b]++, peptide binds strongly and can tolerate anion-exchange chromatography.

+-, peptide binds DLA-88*001:04 but cannot tolerate anion-exchange chromatography.

--, peptide does not bind DLA-88*001:04.
